# The Microscopist; or a Complete Manual on the Use of the Microscope; for Physicians, Students, and All Lovers of Natural Science

**Published:** 1852-01

**Authors:** 


*The Microscopist; or a Complete Manual on the Use of the Microscope; for Physicians, Students, and all Lovers of Natural Science. With Illustrations.* By JOSEPH H. WYTHES, M. D., Phil.; Lindsay and Blackiston.

This is a well printed duodecimo volume, of not quite two hundred pages, It is intended, as the author states in his preface, to serve as a guide to those desirous of applying themselves to microscopic research. The later and more complete English works on the subject are expensive and difficult of access; and as no original American manual has heretofore appeared, Dr. Wythes' volume is an attempt to supply the deficiency. It gives, in a condensed and comparatively cheap form, various directions which will be found highly useful to beginners in microscopic studies. In particular, the chapter on "Procuring Objects for the Microscope," and that on "Minute Injections," contain many valuable hints. As a whole, however, the book has serious deficiencies. It cannot be said to be either elementary or complete. In the chapter on the several varieties of the microscope, an instrument manufactured by Smith & Beck, of London, is described at length, but no allusion is made to those of Nachet, at Paris, or to the American instruments manufactured by Mr. Spencer. Nachet's microscopes, certainly, cannot be said to be "far inferior to the English;" and those of Spencer are acknowledged, on all hands, to be fully equal, if not superior, to those of any other market.

In the chapters on "The Cell-doctrine," "Morbid Structures," and "Urinary Deposits," liberal extracts have been made from foreign works. These extracts, however, are not sufficiently copious to supply the place of the treatises from which they were taken. A book can hardly claim to be a "Complete Manual on the Use of the Microscope," in which the description of muscular fibre occupies only half a page, and the red corpuscles of the blood are disposed of in fifteen lines.

We observe one verbal inaccuracy recurring several times in the course of the book, which is also to be met with in other medical treatises. *An animalcula* may be English, but it certainly is not Latin. Animalcula is the plural of animalculum. A more serious error occurs in the chapter on Urinary Deposits, in which it is stated that uric acid "combines with the alkalies, forming salts which are *insoluble, or very sparingly soluble in water.*" With regard to the microscopic appearances of Morbid structures, we cannot do better than to quote from the author himself: "For further observations on microscopic pathology, the reader is referred to Vogel's Pathological Anatomy, and other similar works." J. C. D.

